# Fatal Delayed-Onset Overwhelming Post-splenectomy Infection 26 Years After Splenectomy for Immune Thrombocytopenic Purpura

**DOI:** 10.7759/cureus.111184

**Published:** 2026-06-19

**Authors:** David Ramzy, Rouba Isshak, Katrina Villegas, Aqsa Sorathia, Arielle H Aiken, Mourad Ismail

**Affiliations:** 1 Internal Medicine, St. Joseph's Regional Medical Center, Paterson, USA; 2 Pulmonary and Critical Care, St. Joseph's Regional Medical Center, Paterson, USA

**Keywords:** asplenia, disseminated intravascular coagulation, encapsulated organisms, immune thrombocytopenic purpura, opsi, overwhelming post-splenectomy infection, pneumococcal vaccination, purpura fulminans, septic shock, streptococcus pneumoniae

## Abstract

Overwhelming post-splenectomy infection (OPSI) is a rare but life-threatening complication that can occur at any time after splenectomy, most often involving encapsulated organisms such as *Streptococcus pneumoniae*, *Neisseria meningitidis*, and *Haemophilus influenzae*.

We report the case of a 36-year-old Caucasian female, 26 years post-splenectomy for immune thrombocytopenic purpura, who presented with flu-like symptoms and altered mentation. On arrival, she was hypotensive, tachycardic, febrile, and hypoglycemic with purpura fulminans and conjunctival injection. She rapidly progressed to septic shock requiring vasopressors, disseminated intravascular coagulation, and acute respiratory failure necessitating mechanical ventilation. Blood cultures revealed *Streptococcus pneumoniae* bacteremia, and the pneumonia panel identified non-COVID-19 coronavirus, adenovirus, *Haemophilus influenzae*, and *Staphylococcus aureus*. Her course was complicated by acute kidney injury requiring continuous renal replacement therapy and bilateral lower limb ischemia. Despite aggressive management, she deteriorated rapidly and died within 48 hours of admission.

This case underscores the critical nature of OPSI and purpura fulminans in asplenic patients and emphasizes the importance of prompt recognition and treatment. While vaccination against encapsulated organisms reduces risk, vaccination failures remain possible. Thorough patient education regarding prodromal symptoms and the integration of prophylactic measures, including early antibiotic initiation, are essential. The high mortality associated with OPSI, even decades after splenectomy, highlights the necessity of ongoing preventive strategies, vigilant monitoring, and rapid intervention to mitigate fatal outcomes.

## Introduction

Overwhelming post-splenectomy infection (OPSI) is a rapidly progressive and often fatal condition in asplenic individuals, typically caused by encapsulated organisms such as *Streptococcus pneumoniae*, *Haemophilus influenzae*, and *Neisseria meningitidis* [1]. Although uncommon, OPSI carries an estimated lifetime risk of approximately 5% and a reported mortality of 40-70%, reflecting its fulminant course and narrow therapeutic window [1,2]. Although the risk is highest within the first few years after splenectomy, cases occurring decades later have been reported, underscoring the lifelong vulnerability of these patients; cohort data confirm persistent risk and frequent gaps in preventive care [3-5].

The spleen is central to the clearance of encapsulated bacteria and to mounting an effective humoral immune response [1,2]. In its absence, patients are predisposed to severe infections that can progress to purpura fulminans, disseminated intravascular coagulation (DIC), and multiorgan failure [4,6]. Early recognition and prompt antimicrobial therapy improve outcomes, but mortality remains high, particularly when treatment is delayed [6].

Prevention through vaccination, patient education, and, in selected cases, antibiotic prophylaxis is essential; however, adherence to recommended guidelines remains suboptimal worldwide [7,8]. This case illustrates the catastrophic consequences of OPSI and underscores the importance of sustained preventive strategies and vigilance, regardless of the time elapsed since splenectomy.

## Case presentation

A 36-year-old Caucasian woman with a history of immune thrombocytopenic purpura (ITP) treated with splenectomy at age 10 presented to the emergency department (ED) after being found unresponsive at home. Two days before presentation, she developed upper respiratory symptoms - low-grade fever, headache, myalgia, arthralgia, and generalized weakness - which progressed to nausea, non-bloody non-bilious vomiting, and a purpuric rash involving the trunk and extremities. On the day of presentation, she developed altered mentation, lethargy, shortness of breath, and a worsening rash, prompting activation of emergency medical services (EMS). Her vaccination history was unknown, was not available at the time of admission, and remained unavailable throughout her hospitalization.

On EMS arrival, she was hypoxic, hypotensive, and profoundly hypoglycemic (glucose 28 mg/dL), for which she received two ampules of dextrose 10% (D10). In the ED, she was hypotensive (requiring vasopressor support), tachycardic, febrile, and hypoglycemic, and was obtunded with altered mentation; a formal Glasgow Coma Scale (GCS) score was not documented on arrival. Examination revealed an extensive purpuric rash involving the face, chest, abdomen, and lower extremities, with bilateral conjunctival injection.

Laboratory studies revealed severe thrombocytopenia (platelet count 27 × 10⁹/L) and profound lactic acidosis (lactate 12.9 mmol/L); the initial arterial blood gas (ABG) showed a pH of 7.19, pCO₂ of 26 mmHg, and bicarbonate of 9.9 mmol/L (Table 1). She was resuscitated with intravenous lactated Ringer's solution (2.5 L in the ED and a further 2 L in the medical intensive care unit (ICU)), a D10 bolus for hypoglycemia, intravenous hydrocortisone (50 mg every six hours), an antihistamine, and empiric broad-spectrum antibiotics (cefepime, azithromycin, clindamycin, and daptomycin; rationale below).

**Table 1:** Laboratory findings at presentation. The glucose value is the prehospital (EMS) measurement; complete blood count and chemistry results represent the most abnormal value recorded within the first 24 hours of admission. Reference ranges are typical adult values and vary by laboratory. Findings are consistent with DIC, severe tissue hypoperfusion, and ischemic hepatic injury. ↑, above reference range; ↓, below reference range; Critical, critical (panic) value; *hypocapnia reflects respiratory compensation for the severe metabolic (lactic) acidosis. ALT: alanine aminotransferase; AST: aspartate aminotransferase; EMS: emergency medical services; FEU: fibrinogen equivalent units; HCO₃⁻: bicarbonate; INR: international normalized ratio; LDH: lactate dehydrogenase; pCO₂: partial pressure of carbon dioxide

Parameter	Patient value	Reference range	Flag
Metabolic			
Glucose (on EMS arrival)	28 mg/dL	70-99 mg/dL	Critical ↓
Lactate	12.9 mmol/L	0.5-2.2 mmol/L	Critical ↑
Calcium (total)	6.0 mg/dL	8.5-10.5 mg/dL	Critical ↓
Renal			
Blood urea nitrogen	31 mg/dL	7-20 mg/dL	↑
Creatinine	2.37 mg/dL	0.6-1.1 mg/dL	↑
Hepatic			
AST	8,700-11,600 U/L	10-40 U/L	Critical ↑
ALT	3,300-4,000 U/L	7-56 U/L	Critical ↑
LDH	1,700 U/L	140-280 U/L	↑
Total bilirubin	2.6-2.8 mg/dL	0.1-1.2 mg/dL	↑
Total protein	3.6-4.8 g/dL	6.0-8.3 g/dL	↓
Albumin	2.4-3.0 g/dL	3.5-5.0 g/dL	↓
Arterial blood gas			
pH	7.19	7.35-7.45	↓
pCO₂	26 mmHg	35-45 mmHg	↓*
Bicarbonate (HCO₃⁻)	9.9 mmol/L	22-26 mmol/L	↓
Hematology/coagulation			
White blood cell count	16.4 × 10⁹/L	4.5-11.0 × 10⁹/L	↑
Differential	Neutrophilia with left shift (bandemia; metamyelocytes and myelocytes present)	-	-
Platelet count	27 × 10⁹/L	150-400 × 10⁹/L	Critical ↓
INR	8.1	0.8-1.1	Critical ↑
Fibrinogen	60 mg/dL	200-400 mg/dL	↓
D-dimer	>20,000 ng/mL FEU	<500 ng/mL FEU	Critical ↑
Peripheral blood smear	Decreased platelets; rare schistocytes; moderate burr cells (echinocytes)	Normal morphology	-
Microbiology			
Blood culture	Streptococcus pneumoniae	No growth	Positive
Respiratory pathogen panel	Non-COVID-19 coronavirus, adenovirus, *Haemophilus influenzae*, *Staphylococcus aureus*	Negative	Positive

The antimicrobial regimen comprised cefepime (2 g every 12 hours), clindamycin (900 mg every six hours), daptomycin (500 mg every 24 hours), and a single dose of azithromycin (500 mg). Cefepime provided broad gram-negative and pneumococcal coverage for suspected severe community-acquired infection, while daptomycin was selected for concern of invasive gram-positive bacteremia and possible severe skin and soft-tissue infection in the setting of purpura fulminans. Clindamycin was added for its antitoxin (toxin-suppressing) properties in suspected streptococcal or staphylococcal toxic shock syndrome. Azithromycin was given empirically as a single dose for possible rickettsial infection, as a doxycycline allergy precluded first-line tetracycline therapy.

She was admitted to the medical ICU for septic shock, hypoxic respiratory failure, and suspected DIC, and was intubated for worsening respiratory distress and hypoxia. Portable chest radiography demonstrated increased bilateral interstitial markings with small bilateral pleural effusions and the endotracheal tube tip at the carina (Figure 1). Non-contrast computed tomography (CT) of the chest, abdomen, and pelvis showed moderate pulmonary edema with dependent ground-glass opacities and small bilateral pleural effusions (Figure 2); the spleen was not visualized, consistent with the prior splenectomy. Laboratory findings supported DIC, with an INR of 8.1, fibrinogen of 60 mg/dL, and D-dimer >20,000 ng/mL. Hepatic markers were markedly abnormal, with aspartate aminotransferase (AST) 8,700-11,600 U/L, alanine aminotransferase (ALT) 3,300-4,000 U/L, lactate dehydrogenase (LDH) 1,700 U/L, total bilirubin 2.6-2.8 mg/dL, and hypoalbuminemia (albumin 2.4-3.0 g/dL), a pattern consistent with ischemic hepatitis (Table 1).

**Figure 1:**
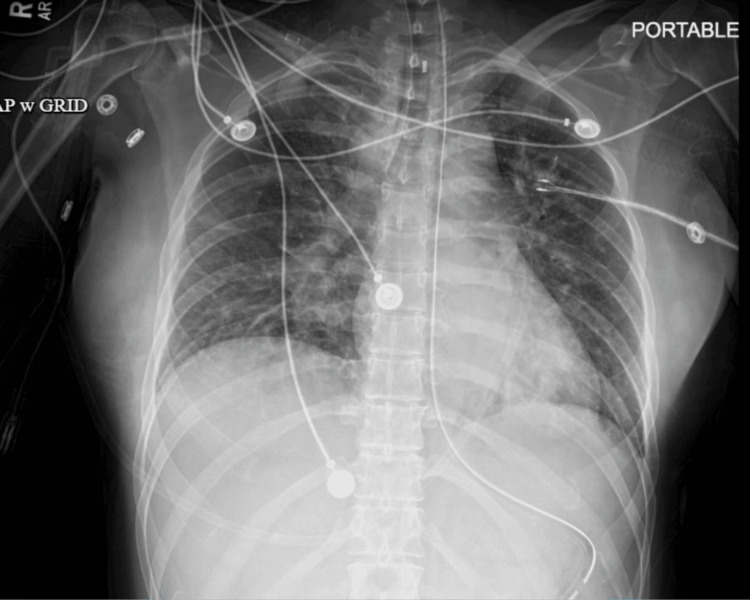
Portable anteroposterior chest radiograph. Increased bilateral interstitial and bronchovascular markings with small bilateral pleural effusions, consistent with pulmonary edema; superimposed pneumonia could not be excluded. The endotracheal tube tip projects at the level of the carina (superior repositioning was recommended); support lines and ECG leads are also seen.

**Figure 2:**
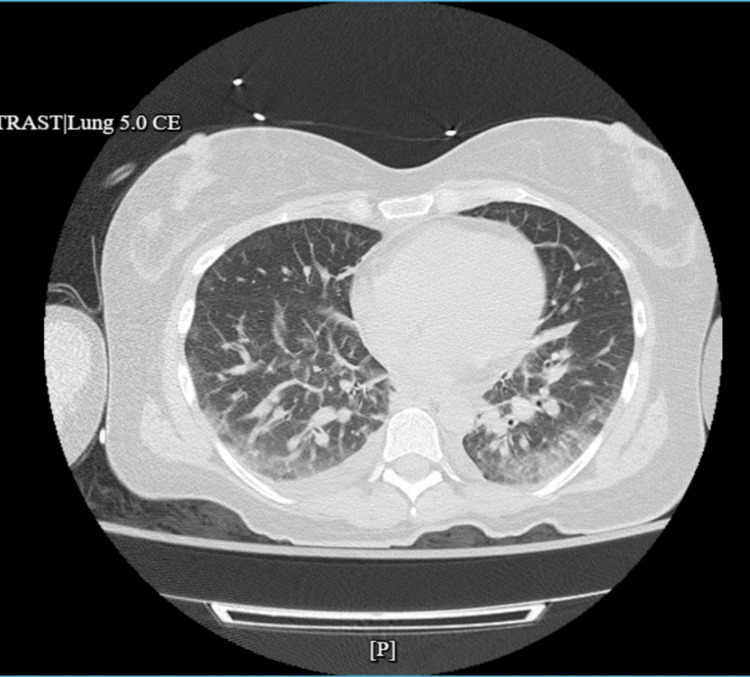
Axial chest CT (lung window, without intravenous contrast). Bilateral, dependently distributed ground-glass opacities with interlobular septal thickening, most pronounced in the lower lobes, consistent with moderate pulmonary edema; superimposed pneumonia could not be excluded. Small bilateral pleural effusions were also present.

Blood cultures grew *Streptococcus pneumoniae*. A respiratory pathogen panel, obtained from a deep tracheal aspirate, detected non-COVID-19 coronavirus, adenovirus, *Haemophilus influenzae*, and *Staphylococcus aureus*.

Despite antimicrobial therapy and supportive care, her course was complicated by acute kidney injury (AKI) requiring continuous renal replacement therapy (CRRT). Hematology was consulted to exclude thrombotic thrombocytopenic purpura (TTP); the peripheral blood smear showed thrombocytopenia with only rare schistocytes and moderate burr cells (echinocytes), insufficient to support a microangiopathic hemolytic process and thereby arguing against TTP. Platelet transfusion was administered despite the absence of overt bleeding because the combination of severe thrombocytopenia (27 × 10⁹/L) and active DIC with purpura fulminans conferred a high hemorrhagic risk, and to support invasive procedures, including endotracheal intubation and central venous access.

On hospital day 3, she developed worsening acidosis, hyperkalemia, and bilateral lower-limb ischemia confirmed on arterial Doppler. Despite aggressive resuscitative efforts, she suffered cardiopulmonary arrest and died.

## Discussion

We present a 36-year-old woman who developed fatal OPSI with septic shock and multiorgan failure 26 years after splenectomy for ITP. Despite prompt recognition and aggressive treatment - broad-spectrum antibiotics, CRRT, and intensive supportive care - she deteriorated rapidly and died.

The spleen is central to host defense against encapsulated organisms such as *Streptococcus pneumoniae*, *Haemophilus influenzae*, and *Neisseria meningitidis*, filtering circulating pathogens, presenting antigens, and producing opsonins such as IgM; asplenia impairs bacterial clearance and predisposes to fulminant bacteremia and OPSI, a medical emergency with high mortality that demands immediate recognition and intervention [1].

Although OPSI risk is highest in the first two years after splenectomy, it never fully resolves [3,9]. A literature review estimated an annual serious-infection incidence of 0.23% and a lifetime OPSI risk of approximately 5%, influenced by age, splenectomy indication, and immunosuppression, with the reported interval between splenectomy and OPSI ranging from less than one week to several decades [2]. Illustrative reports span this range: Abe et al. described *Escherichia coli* purpura fulminans 20 years after splenectomy in a patient receiving corticosteroids [3]; Ito et al. reported pneumococcal prosthetic-valve endocarditis 30 years after splenectomy in an unvaccinated patient [10]; and Gupta and Vazquez described fatal OPSI within 24 hours of symptom onset, 15 years after splenectomy [11]. Notably, several of these cases carried additional predisposing factors absent in our patient - corticosteroid immunosuppression in the report by Abe et al. [3] and a prosthetic valve, an independent risk factor for endocarditis, in the report by Ito et al. [10] - whereas our patient had no chronic immunosuppression, prosthetic material, or other identified risk factor beyond long-standing asplenia. Fatal pneumococcal OPSI has nonetheless occurred even in vaccinated asplenic adults [12]. Taken together, our case at 26 years reinforces that no interval since splenectomy confers immunity, and that lifelong vigilance - continued patient education, vaccination adherence, and a low threshold for empiric broad-spectrum therapy - is warranted in any febrile asplenic patient.

OPSI is typically nonspecific at onset - fever, malaise, or myalgia - progressing within hours to septic shock, DIC, and multiorgan failure, with purpura fulminans, a hallmark manifestation, reflecting microvascular thrombosis and consumptive coagulopathy [3,4]. In our patient, the combination of marked thrombocytopenia and coagulopathy (INR 8.1, hypofibrinogenemia, and a markedly elevated D-dimer) reflected DIC, while the severe lactic acidosis indicated profound tissue hypoperfusion. The accompanying hypoglycemia is not itself a feature of DIC; rather, it is a marker of severe sepsis with hepatic dysfunction, as hepatic gluconeogenesis and lactate clearance become overwhelmed in fulminant infection. This is supported by markedly elevated transaminases (AST 8,700-11,600 U/L and ALT 3,300-4,000 U/L) with an elevated LDH (1,700 U/L) and hyperbilirubinemia - a pattern characteristic of ischemic (hypoxic) hepatitis, or “shock liver,” arising from hepatic hypoperfusion in profound shock - while the concurrent hypoalbuminemia and low total protein reflect impaired hepatic synthetic function. Such fulminant hepatic injury impairs gluconeogenesis and lactate clearance, accounting for both the hypoglycemia and the severity of the lactic acidosis. Collectively, these findings indicated severe, rapidly evolving multiorgan compromise and underscored the need to recognize subtle early symptoms and respond promptly.

Our patient's vaccination status could not be ascertained at any point during her admission; consequently, it was not possible to determine whether her infection represented vaccine failure or occurred in an unvaccinated host. This distinction is clinically meaningful, as fatal pneumococcal OPSI has been documented even in appropriately vaccinated asplenic adults [12], reinforcing that vaccination reduces but does not eliminate risk.

Diagnosis is often delayed because early symptoms are nonspecific and asplenic status may be overlooked, particularly when splenectomy was remote [1,4,9]; even brief delays can be fatal. Given the potential for resistant or polymicrobial infection, early collaboration with infectious diseases and microbiology is essential. Per current American Thoracic Society (ATS)/Infectious Diseases Society of America (IDSA) guidance, empiric therapy for severe community-acquired pneumonia (CAP) combines a beta-lactam with a macrolide or a respiratory fluoroquinolone, adding coverage for methicillin-resistant *Staphylococcus aureus* (MRSA) or *Pseudomonas aeruginosa* when risk factors are present [13]; early aggressive antimicrobials combined with intensive care can reduce OPSI mortality to as low as 10% [14]. Our patient's prominent respiratory symptoms and hypoxic respiratory failure made severe CAP a leading diagnosis.

The initial regimen - cefepime, azithromycin, daptomycin, and clindamycin - was chosen to cover typical respiratory and gram-negative pathogens, invasive gram-positive bacteremia, possible toxin-mediated disease in the setting of purpura fulminans and shock, and empiric atypical or rickettsial coverage given a doxycycline allergy; daptomycin and clindamycin were added empirically before culture data. Notably, daptomycin is inappropriate for pneumonia because it is inactivated by pulmonary surfactant; in retrospect, strict guideline-directed CAP therapy would have been preferable, highlighting the importance of evidence-based empiric selection in the early hours of sepsis. Despite prompt broad-spectrum therapy, the outcome reflects the fulminant nature of OPSI and its narrow therapeutic window, with an estimated mortality of 40-70% [1].

Because established OPSI is difficult to treat, prevention is paramount. Immunization against *Streptococcus pneumoniae*, *Haemophilus influenzae* type b (Hib), *Neisseria meningitidis* (MenACWY and MenB), and influenza, with lifelong booster dosing, is essential [1,7,15]. Antibiotic prophylaxis may benefit high-risk groups, such as young children, older adults, and those with prior OPSI, but the decision must balance the protective benefit of prophylaxis against the risk of antimicrobial resistance, particularly given the availability of newer vaccines [10]; the supporting evidence derives largely from pediatric sickle-cell populations and is weak for adult splenectomy, leaving routine use uncertain [16]. Patient education is equally critical: recognizing early infection and carrying both medical identification and emergency standby antibiotics can be lifesaving [15], with standby-antibiotic prescribing individualized to the patient's risk [8,10,17].

Optimal care also requires a multidisciplinary approach - here, coordination among critical care, infectious diseases, nephrology, and hematology - together with attention to systemic gaps in long-term follow-up, as many patients and providers underappreciate the lifelong risk, especially after childhood or trauma-related splenectomy. Structured discharge education, vaccine tracking, and primary-care reminders are essential, particularly in underserved populations, and further research is needed to refine prevention and treatment [1]. Ultimately, this case reflects the devastating speed of OPSI, which can outpace optimal care even in a high-resource setting, and underscores the importance of early suspicion in any febrile asplenic patient.

## Conclusions

OPSI remains a life-threatening condition in asplenic patients, with mortality that stays high despite advances in medical care. This case highlights the critical importance of preventive strategies, including timely pneumococcal, Hib, meningococcal, and annual influenza vaccination, and antibiotic prophylaxis for selected asplenic individuals. Early recognition and prompt initiation of appropriate antimicrobial therapy are essential to improving outcomes, particularly in cases complicated by sepsis and multiorgan dysfunction. The complexity of this patient's presentation further underscores the need for a multidisciplinary approach - involving critical care, infectious diseases, hematology, and nephrology - to address complications such as DIC, AKI, and peripheral ischemia. Public health efforts to enhance patient education and improve vaccination access are crucial to reducing the incidence and severity of OPSI.

## References

[1] Tahir F, Ahmed J, Malik F (2020). Post-splenectomy sepsis: a review of the literature. Cureus.

[2] Sinwar PD (2014). Overwhelming post splenectomy infection syndrome - review study. Int J Surg.

[3] Abe Y, Itagaki H, Endo T (2023). Overwhelming post-splenectomy infection caused by Escherichia coli 20 years after splenectomy: a case report. Cureus.

[4] Komatsu M, Ishihara T, Manabe S (2017). A case of overwhelming postsplenectomy infection caused by Streptococcus pneumoniae with fulminant purpura. Tokai J Exp Clin Med.

[5] Soderstrom MA, Miller MA, Wang Q (2024). Vaccine utilization and overwhelming post-splenectomy infection risk factors in two asplenia cohorts. Haematologica.

[6] Evans L, Rhodes A, Alhazzani W (2021). Surviving sepsis campaign: international guidelines for management of sepsis and septic shock 2021. Intensive Care Med.

[7] Orangzeb S, Watle SV, Caugant DA (2023). Adherence to vaccination guidelines of patients with complete splenectomy in Norway, 2008-2020. Vaccine.

[8] Bianchi FP, Stefanizzi P, Spinelli G, Mascipinto S, Tafuri S (2021). Immunization coverage among asplenic patients and strategies to increase vaccination compliance: a systematic review and meta-analysis. Expert Rev Vaccines.

[9] Chong J, Jones P, Spelman D, Leder K, Cheng AC (2017). Overwhelming post-splenectomy sepsis in patients with asplenia and hyposplenia: a retrospective cohort study. Epidemiol Infect.

[10] Ito T, Yokota K, Yamamoto S (2025). Prosthetic double valve endocarditis caused by Streptococcus pneumoniae due to overwhelming post-splenectomy infection (OPSI) in a pre-vaccine licensure era: a case report. J Infect Chemother.

[11] Gupta AK, Vazquez OA (2020). Overwhelming post-splenectomy infection syndrome: variability in timing with similar presentation. Cureus.

[12] Santunione AL, Camatti J, Zucchi F (2025). Fatal Waterhouse-Friderichsen syndrome caused by Streptococcus pneumoniae in a vaccinated adult with traumatic splenectomy: a case report. Leg Med (Tokyo).

[13] Metlay JP, Waterer GW, Long AC (2019). Diagnosis and treatment of adults with community-acquired pneumonia. An official clinical practice guideline of the American Thoracic Society and Infectious Diseases Society of America. Am J Respir Crit Care Med.

[14] Morgan TL, Tomich EB (2012). Overwhelming post-splenectomy infection (OPSI): a case report and review of the literature. J Emerg Med.

[15] Luu S, Spelman D, Woolley IJ (2019). Post-splenectomy sepsis: preventative strategies, challenges, and solutions. Infect Drug Resist.

[16] Lau JS, Korman TM, Woolley I (2018). Life-long antimicrobial therapy: where is the evidence?. J Antimicrob Chemother.

[17] Davidson RN, Wall RA (2001). Prevention and management of infections in patients without a spleen. Clin Microbiol Infect.

